# Prof. Meigs on Females and Their Diseases

**Published:** 1848-02

**Authors:** 


### PROF. MEIGS ON FEMALES AND THEIR DISEASES.

The Boston Medical and Surgical Journal is warm in its praises of this new work. The editor remarks of it: "We relished the style—a flowing, conversational freedom of thought and expression characterizing the whole, and giving even pretty grave parts an inviting aspect. The fact could not be denied, as we thought, that the volume was well calculated to have an extensive circulation, on account of the precepts it inculcates, the practical value it really possesses, and the lively, agreeable method Dr. Meigs has discovered of gaining the entire attention of the reader."

A communication in the same Journal, signed with the initials of the distinguished Professor of Obstetrics in the Boston school, pronounces it "a clever, a very clever work. It is *unique* in its method, and truly felicitous in its execution."

